# Present to Dr. Morton

**Published:** 1848-08

**Authors:** 


*Present to Dr. Morton.*—A silver box, containing one thousand dollars, has been presented to Dr. Morton, with the following inscription:—"This box, containing $1000, is presented to Wm. Thos. Green Morton, by the members of the Board of Trustees of the Mass. Gen. Hospital, and other citizens of Boston, May 3d, 1848. He has become poor in a cause in which he has made the world his debtor. Testimonial in honor of the ether discovery of Sept. 30. 1046."

Our friend of the Annalist remarks, that this settles his claim to the discovery. We hope Dr. Jackson's friends will not allow themselves to be outdone by evidence of this nature.

